# Association of MYLIP rs3757354 SNP and several environmental factors with serum lipid levels in the Guangxi Bai Ku Yao and Han populations

**DOI:** 10.1186/1476-511X-11-141

**Published:** 2012-10-29

**Authors:** Ting-Ting Yan, Rui-Xing Yin, Qing Li, Ping Huang, Xiao-Na Zeng, Ke-Ke Huang, Dong-Feng Wu, Lynn Htet Htet Aung

**Affiliations:** 1 Department of Cardiology, Institute of Cardiovascular Diseases, the First Affiliated Hospital, Guangxi Medical University, University, 22 Shuangyong Road, Nanning 530021, Guangxi, People’s Republic of China

## Abstract

**Background:**

The association of rs3757354 single nucleotide polymorphism (SNP) in the E3 ubiquitin ligase myosin regulatory light chain-interacting protein (MYLIP, also known as IDOL) gene and serum lipid levels is not well known in the general population. The present study aimed to detect the association of rs3757354 SNP and several environmental factors with serum lipid levels in the Guangxi Bai Ku Yao and Han populations.

**Method:**

A total of 627 subjects of Bai Ku Yao minority and 614 participants of Han nationality were randomly selected from our stratified randomized cluster samples. Genotyping of the rs3757354 SNP was performed by polymerase chain reaction and restriction fragment length polymorphism combined with gel electrophoresis, and then confirmed by direct sequencing.

**Results:**

The levels of serum total cholesterol (TC), high-density lipoprotein cholesterol (HDL-C), low-density lipoprotein cholesterol (LDL-C), apolipoprotein (Apo) AI and ApoB were lower in Bai Ku Yao than in Han (*P* < 0.05-0.001). The frequency of G allele was 49.92% in Bai Ku Yao and 56.27% in Han (*P* < 0.05). The frequencies of AA, GA and GG genotypes were 25.52%, 49.12% and 25.36% in Bai Ku Yao, and 19.87%, 47.72% and 32.41% in Han (*P <* 0.05); respectively. There were no significant differences in the genotypic and allelic frequencies between males and females in both ethnic groups. The levels of HDL-C in Bai Ku Yao were different among the genotypes (*P* < 0.05), the G allele carriers had higher serum HDL-C levels than the G allele noncarriers. The levels TC, HDL-C and ApoAI in Han were different among the genotypes (*P* < 0.05 for all), the participants with GA genotype had lower serum TC, HDL-C and ApoAI levels than the participants with AA genotype. These findings were found only in females but not in males. The levels of TG and HDL-C in Bai Ku Yao were correlated with the genotypes, whereas the levels of TC in Han, and TC, LDL-C in Han females were associated with the genotypes (*P* < 0.05 for all). Serum lipid parameters were also correlated with age, sex, alcohol consumption, cigarette smoking, blood pressure, and body mass index in both ethnic groups (*P* < 0.05-0.001).

**Conclusions:**

The present study suggests that the MYLIP rs3757354 SNP is associated with serum TC, HDL-C and ApoAI levels in the Bai Ku Yao and Han populations. But the association is different between the two ethnic groups.

## Introduction

Many epidemiological and clinical studies have shown that dyslipidemia is strongly associated with an increased risk of coronary artery disease (CAD) [1-5]. It is generally agreed that dyslipidemia is a complex trait caused by genetic factors and multiple environmental risk factors such as diet, alcohol consumption, cigarette smoking, obesity, physical inactivity, hypertension and their interactions [6-9]. Family studies have shown that in many populations, genetic polymorphisms could account for half of the variation in serum lipid phenotypes [10,11], and it is clear that serum lipid levels are strongly influenced by the genetic constitution of each individual.

Recent genome-wide association studies (GWAS) in different populations have identified more than 95 loci influence plasma lipid levels [12-23]. These studies evaluated large samples of normolipidemic individuals and showed that several new single nucleotide polymorphisms (SNPs) had replicable modest associations with plasma concentrations of total cholesterol (TC), triglyceride (TG), low-density lipoprotein cholesterol (LDL-C), and high-density lipoprotein cholesterol (HDL-C) [15-18]. In addition, GWAS also discovered a number of novel loci that influence serum lipid phenotypes [23-25]. One of these newly identified SNPs is the E3 ubiquitin ligase myosin regulatory light chain-interacting protein (MYLIP, also known as IDOL) gene. MYLIP locates on chromosome 6p22.3 which stimulates ubiquitination of the low-density lipoprotein receptor (LDLR) on its cytoplasmic tail, thereby directing its degradation [24]. It is also known as post-transcriptional regulator of LDLR abundance [26-33]. The *LXR-MYLIP-LDLR* pathway provides a complementary pathway to sterol regulatory element-binding proteins for the feedback inhibition of cholesterol uptake [34]. Several SNPs including MYLIP rs9370867, rs3757354 and rs2327951 have been associated with human plasma cholesterol [35]. However, the rs3757354 SNP has not been reported in prior GWAS for plasma lipid concentration [24]. Teslovich *et al*. [23] demonstrated 95 loci that showed genome-wide significant association with blood lipid, and rs3757354 was shown significant association with LDL-C and TC. Weissglas-Volkov *et al*. [35] demonstrated that N342S in MYLIP was associated with higher TC levels in Mexican dyslipidemic individuals. However, Santos *et al*. [36] involved MYLIP p.N342S polymorphism is not associated with lipid profiles in the Brazilian population and indicated that further studies are needed to reaffirm the MYLIP polymorphism function. Besides, Banka *et al*. [37] and Kararigas *et al*. [38] reported that MYLIP is a sex-specific element influencing contractile function. Thus, it would be interesting to characterize the full impact of the relationship between rs3757354 and serum lipid levels.

China is a multiethnic country. There are 56 ethnic groups. Han nationality is the largest ethnic group, and Yao nationality is the eleventh largest minority among the 55 minority groups according to the population size. Bai Ku Yao (White-trouser Yao), an isolated subgroup of Yao nationality, is named so because all the men wear white knee-length knickerbockers. The population size is about 30000. Because of isolation from the other ethnic groups, the special customs and cultures including their clothing, intra-ethnic marriages, ballad, funeral, bronze drum, spinning top, dietary habits, and corn wine and rum intakes are still completely preserved to the present day. In several previous studies, we found that the serum lipid concentrations were lower in Bai Ku Yao than in Han from the same region [7,8,39]. This ethnic difference in serum lipid profiles is still unknown. We hypothesized that there may be significant differences in some genetic polymorphisms between the two ethnic groups [40-44]. Therefore, the aim of the present study was to detect the association between rs3757354 SNP in the MYLIP gene and several environmental factors with serum lipid profiles in the Guangxi Bai Ku Yao and Han populations.

## Materials and methods

### Participants

The participants in the present study included 627 individuals of Bai Ku Yao who live in Lihu and Baxu villages in Nandan County, Guangxi Zhuang Autonomous Region, People’s Republic of China. They were randomly selected from our previous stratified randomized cluster samples [7]. The ages of the participants ranged from 17 to 80 years, with an average age of 40.59 ± 14.21 years. There were 303 males (48.3%) and 324 females (51.7%). All participants were rural agricultural workers. During the same period, a total of 614 people of Han nationality who reside in the same villages were also randomly selected from our previous stratified randomized cluster samples. The mean age of the subjects was 40.94 ± 15.56 years (range 15 to 86). There were 294 men (47.9%) and 320 women (52.1%). All of them were also rural agricultural workers. All study subjects were essentially healthy and had no evidence of any chronic illness, including hepatic, renal, or thyroid. The participants with a history of heart attack or myocardial infarction, stroke, congestive heart failure, diabetes or fasting blood glucose ≥ 7.0 mmol/L determined by glucose meter were excluded from the analyses. The participants were not taking medications known to affect serum lipid levels (lipid-lowering drugs such as statins or fibrates, beta-blockers, diuretics, or hormones). The experimental design was approved by the Ethics Committee of the First Affiliated Hospital, Guangxi Medical University. All participants in this study had provided written informed consent.

### Epidemiological survey

The survey was carried out using internationally standardized methods, following a common protocol [45]. All participants underwent a complete history, physical examination, and laboratory assessment of cardiovascular risk factors, including lifestyle factors, family history of myocardial infarction, hypertension and diabetes mellitus. Information on demographics, socioeconomic status, and lifestyle factors was collected with standardized questionnaires. The alcohol information included questions about the number of liangs (about 50 g) of rice wine, corn wine, rum, beer, or liquor consumed during the preceding 12 months. Alcohol consumption was categorized into groups of grams of alcohol per day: < 25 and ≥ 25. Smoking status was categorized into groups of cigarettes per day: < 20 and ≥ 20. At the physical examination, several parameters, such as height, weight, and waist circumference were measured. Sitting blood pressure was measured three times with the use of a mercury sphygmomanometer after the subjects had a 5-minute rest, and the average of the three measurements was used for the level of blood pressure. Systolic blood pressure was determined by the first Korotkoff sound, and diastolic blood pressure by the fifth Korotkoff sound. Body weight, to the nearest 50 grams, was measured using a portable balance scale. Subjects were weighed without shoes and in a minimum of clothing. Height was measured, to the nearest 0.5 cm, using a portable steel measuring device. From these two measurements body mass index (BMI, kg/m^2^) was calculated.

### Determination of serum lipid levels

Venous blood samples (5 mL) were collected from all subjects after an overnight (at least 12 hours) fast. A part of the sample (2 mL) was collected into glass tubes and allowed to clot at room temperature, and used to determine serum lipid levels. Another part of the sample (3 mL) was transferred to tubes with anticoagulate solution (4.80 g/L citric acid, 14.70 g/L glucose, and 13.20 g/L tri-sodium citrate) and used to extract DNA. The levels of TC, TG, HDL-C, and LDL-C in the samples were measured according to standard enzymatic methods. Serum apolipoprotein (Apo) AI and ApoB levels were detected by the immunoturbidimetric immunoassay. All determinations were performed with an autoanalyzer (Type 7170A; Hitachi Ltd., Tokyo, Japan) in the Clinical Science Experiment Center of the First Affiliated Hospital, Guangxi Medical University.

### DNA preparation and genotyping

Genomic DNA was isolated from peripheral blood leukocytes using the phenol-chloroform method [40-44]. The extracted DNA was stored at −80°C until analysis. Genotyping of the rs3757354 SNP was performed by polymerase chain reaction and restriction fragment length polymorphism (PCR-RFLP). PCR amplification was performed using 5'-ACAGAGCAAAAGACCCTGTCTC-3' and 5'-AAAGAACTGTGTGTGGGAGGAT-3' (Sangon, Shanghai, People’s Republic of China) as the forward and reverse primer pairs; respectively. Each amplification reaction system of a total volume of 25 μL, comprised 100 ng (2 μL) of genomic DNA; 1.0 μL of each primer (10 μmo1/L); 12.5 μL 2 × *Taq* PCR MasterMix (constituent: 0.1 U *Taq* polymerase/μL, 500 μM dNTP each, 20 mM Tris–HCl, pH 8.3, 100 mM KCl, 3 mM MgCl_2_, and stabilizers), and nuclease-free water 8.5 μL. After initial denaturizing at 95°C for 5 min, the reaction mixture was subjected to 30 cycles of denaturation at 95°C for 30 s, annealing at 61°C for 45 s and extension 1 min at 72°C, followed by a final 5 min extension at 72°C. After electrophoresis on a 1.5% agarose gel with 0.5 μg/mL ethidium bromide, the amplification products were visualized under ultraviolet light. Then 10.0 U of *Hae*III restriction enzyme, 8 μL nuclease-free water and 1 μL of 10 × buffer solution were added directly to the PCR products (5 μL) and digested at 37°C overnight. After restriction enzyme digestion of the amplified DNA, genotypes were identified by electrophoresis on 2.0% agarose gel and visualized with ethidium-bromide staining ultraviolet illumination. Genotypes were scored by an experienced reader blinded to epidemiological data and serum lipid levels.

### DNA sequencing

Six samples (AA, GA and GG genotypes in two; respectively) detected by the PCR-RFLP were also confirmed by direct sequencing. The PCR products were purified by low melting point gel electrophoresis and phenol extraction, and then the DNA sequences were analyzed in Shanghai Sangon Biological Engineering Technology & Services Co., Ltd., People’s Republic of China.

### Diagnostic criteria

The normal values of serum TC, TG, HDL-C, LDL-C, ApoAI, ApoB levels and the ratio of ApoAI to ApoB in our Clinical Science Experiment Center were 3.10–5.17, 0.56–1.70, 1.16–1.42, 2.70–3.20 mmol/L, 1.00–1.78, 0.63–1.14 g/L, and 1.00–2.50; respectively [40]. The individuals with TC > 5.17 mmol/L and/or TG > 1.70 mmol/L were defined as hyperlipidemic [7,8]. Hypertension was diagnosed according to the criteria of 1999 World Health Organization-International Society of Hypertension Guidelines for the management of hypertension [46,47]. The diagnostic criteria of overweight and obesity were according to the Cooperative Meta-analysis Group of China Obesity Task Force. Normal weight, overweight and obesity were defined as a BMI < 24, 24–28, and > 28 kg/m^2^; respectively [48].

### Statistical analysis

Epidemiological data were recorded on a pre-designed form and managed with Excel software. The statistical analyses were done with the statistical software package SPSS 13.0 (SPSS Inc., Chicago, Illinois). Quantitative variables were expressed as mean ± standard deviation (serum TG levels were presented as medians and interquartile ranges). Qualitative variables were expressed as percentages. Allele frequency was determined via direct counting, and the standard goodness-of-fit test was used to test the Hardy-Weinberg equilibrium. Difference in genotype distribution between the groups was obtained using the chi-square test. The difference in general characteristics between two ethnic groups was tested by the Student’s unpaired *t*-test. The association of genotypes and serum lipid parameters was tested by analysis of covariance (ANCOVA). Age, sex, BMI, blood pressure, alcohol consumption, and cigarette smoking were included in the statistical models as covariates. Multiple linear regression analyses adjusted for age, sex, BMI, blood pressure, alcohol consumption, and cigarette smoking were also performed to assess the association of serum lipid levels with genotypes (AA = 1, GA = 2, GG = 3) and several environment factors. A *P* value of less than 0.05 was considered statistically significant.

## Results

### General characteristics and serum lipid levels

The general characteristics and serum lipid levels between the Bai Ku Yao and Han populations are presented in Table 1. The levels of height, weight, systolic blood pressure, pulse pressure, serum TC, HDL-C, LDL-C, ApoAI,and ApoB were lower in Bai Ku Yao than in Han (*P* < 0.05-0.001), whereas the percentage of subjects who smoked cigarettes or consumed alcohol was higher in Bai Ku Yao than in Han (*P* < 0.001). There were no significant differences in the levels of BMI, diastolic blood pressure, serum TG, the ratio of ApoAI to ApoB and the ratio of male to female between the two ethnic groups (*P* > 0.05 for all).

**Table 1:** The general characteristics and serum lipid levels in the Bai Ku Yao and Han populations

**Parameter**	**Bai Ku Yao**	**Han Chinese**	***t *(χ**^**2**^**)**	***P***
Number	627	614	–	–
Male/female	303/324	294/320	0.024	0.910
Age (years)	40.59±14.21	40.94±15.56	−0.425	0.671
Height (cm)	152.70±7.15	154.76±8.22	−4.691	0.000
Weight (kg)	52.05±7.28	54.03±9.58	−4.084	0.000
Body mass index (kg/m^2^)	22.28±2.39	22.50±3.25	−1.349	0.178
Systolic blood pressure (mmHg)	119.69±17.23	121.84±17.76	−2.169	0.030
Diastolic blood pressure (mmHg)	75.94±9.58	76.43±11.26	−0.821	0.412
Pulse pressure (mmHg)	43.75±12.86	45.48±12.27	−2.423	0.016
Cigarette smoking [n (%)]				
Nonsmoker	415 (66.19)	472 (76.87)		
< 20 cigarettes/day	92 (14.67)	64 (10.42)		
≥20 cigarettes/day	120 (19.14)	78 (12.70)	17.463	0.000
Alcohol consumption [n (%)]				
Nondrinker	331 (52.19)	417 (67.91)		
< 25 g/day	189 (30.14)	121 (19.71)		
≥25 g/day	107 (17.07)	76 (12.38)	29.922	0.000
Total cholesterol (mmol/L)	4.38±0.93	4.77±0.99	−7.123	0.000
Triglyceride (mmol/L)	0.99 (0.66)	1.04 (0.74)	−1.605	0.109
HDL-C (mmol/L)	1.70±0.42	1.87±0.48	−6.751	0.000
LDL-C (mmol/L)	2.57±0.78	2.66±0.77	−1.978	0.048
Apolipoprotein (Apo) AI (g/L)	1.33±0.32	1.42±0.27	−5.196	0.000
ApoB (g/L)	0.84±0.23	0.90±0.22	−4.252	0.000
ApoAI/ApoB	1.72±0.80	1.67±0.52	1.331	0.184

*HDL-C*, high-density lipoprotein cholesterol; *LDL-C*, low-density lipoprotein cholesterol. The value of triglyceride was presented as median (interquartile range), the difference between the two ethnic groups was determined by the Wilcoxon-Mann–Whitney test.

### Electrophoresis and genotypes

**Figure 1:**
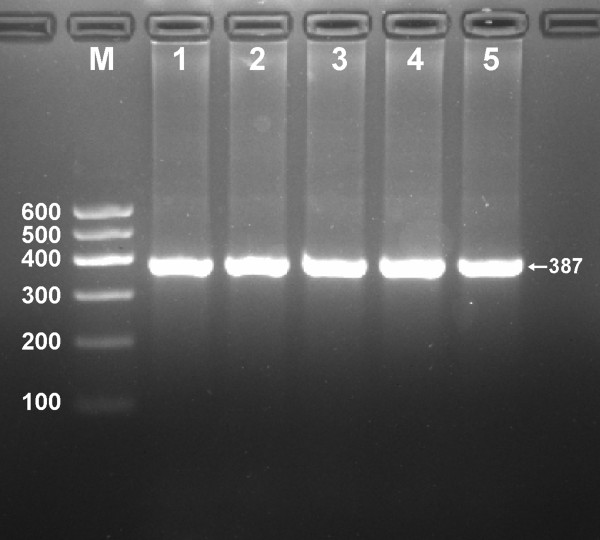
**Electrophoresis of PCR products of the samples. **Lane M, 100 bp marker ladder; lanes 1–5, samples. The 387 bp bands are the target genes.

**Figure 2:**
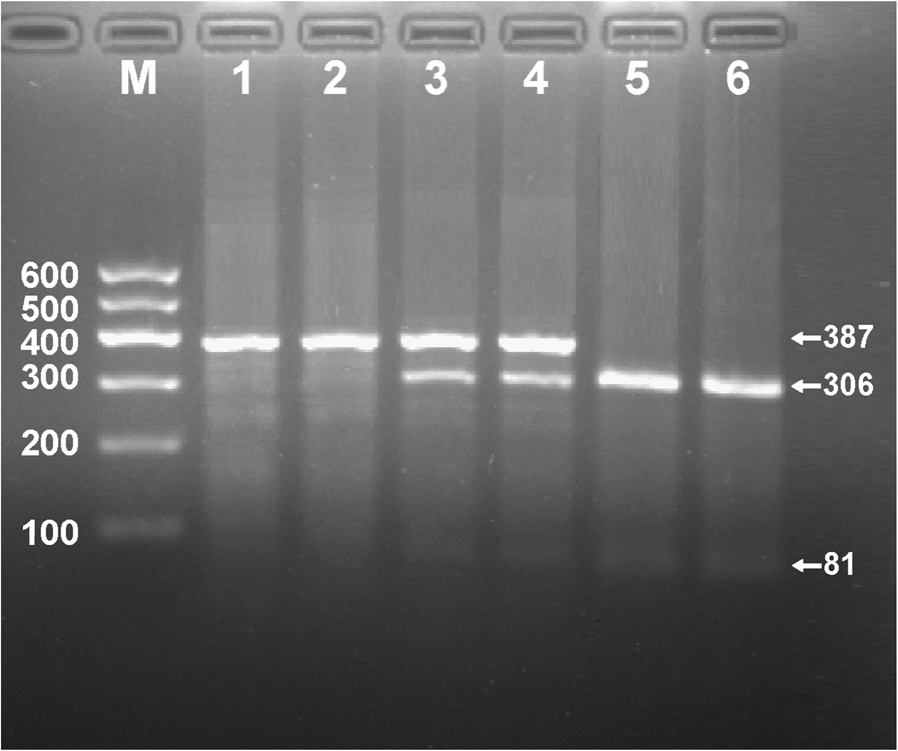
**Genotyping of rs3757354 SNP in **the **MYLIP gene. **Lane M, 100 bp marker ladder; lanes 1 and 2, AA genotype (387 bp); lanes 3 and 4, GA genotype (387-, 306- and 81-bp); and lanes 5 and 6, GG genotype (306- and 81-bp).

After the genomic DNA of the samples was amplified by PCR and imaged by 1.5% agarose gel electrophoresis, the PCR products of 387 bp nucleotide sequences could be seen in the samples (Figure 1). The genotypes identified were named according to the presence or absence of the enzyme restriction sites. The presence of the cutting site indicates the G allele, while its absence indicates the A allele (cannot be cut). Thus, AA genotype is homozygote for the absence of the site (bands at 387 bp), GA genotype is heterozygote for the absence and presence of the site (bands at 387-, 306- and 81-bp), and GG genotype is homozygote for the presence of the site (bands at 306- and 81- bp; Figure 2).

### Nucleotide sequences

**Figure 3:**
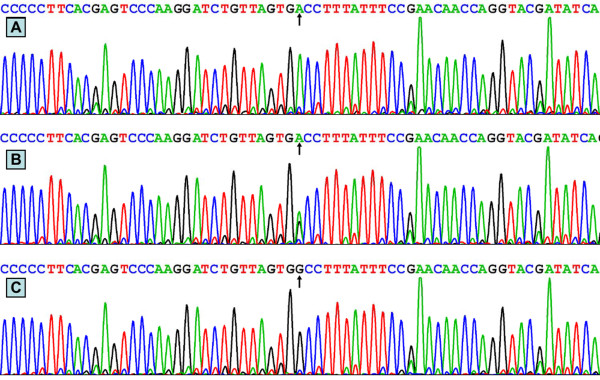
**A part of the nucleotide sequence of rs3757354 SNP. **(**A**) AA genotype; (**B**) GA genotype; (**C**) GG genotype.

The results were shown as AA, GA and GG genotypes of the rs3757354 SNP by PCR-RFLP, the genotypes were also confirmed by sequencing (Figure 3); respectively.

### Genotypic and allelic frequencies

The genotypic and allelic frequencies of rs3757354 SNP in the both ethnic groups are shown in Table 2. The genotype distribution followed the Hardy-Weinberg equilibrium. The A and G allele frequencies were 50.08% and 49.92% in Bai Ku Yao, and 43.73% and 56.27% in Han (*P <* 0.05); respectively. The frequencies of AA, GA and GG genotypes were 25.52%, 49.12% and 25.36% in Bai Ku Yao, and 19.87%, 47.72% and 32.41% in Han (*P <* 0.05); respectively. There were no significant differences in the genotypic and allelic frequencies between males and females in both ethnic groups.

**Table 2:** The genotypic and allelic frequencies of rs3757354 SNP in the Bai Ku Yao and Han populations [n (%)]

**Group**	**n**	**Genotype**			**Allele**	
		**AA**	**GA**	**GG**	**G**	**T**
Bai Ku Yao	627	160 (25.52)	308 (49.12)	159 (25.36)	628 (50.08)	626 (49.92)
Han	614	122 (19.87)	293 (47.72)	199 (32.41)	537 (43.73)	691 (56.27)
χ^2^	–	9.829			10.045	
*P*	–	0.007			0.002	
Bai Ku Yao						
Male	303	75 (24.75)	161 (53.14)	67 (22.11)	311 (51.32)	295 (48.68)
Female	324	85 (26.23)	147 (45.37)	92 (28.40)	317 (48.92)	331 (51.08)
χ^2^	–	4.494			0.722	
*P*	–	0.106			0.397	
Han						
Male	294	61 (20.75)	142 (48.30)	91 (30.95)	264 (44.90)	324 (55.10)
Female	320	61 (19.06)	151 (47.19)	108 (33.75)	273 (42.66)	367 (57.34)
χ^2^	–	0.629			0.626	
*P*	–	0.730			0.454	

### Genotypes and serum lipid levels

As shown in Table 3, the levels of HDL-C in Bai Ku Yao were different among the three genotypes (*P* < 0.05), the G allele carriers had higher serum HDL-C levels than the G allele noncarriers. For the Han population, the levels of TC, HDL-C and ApoAI among the three genotypes were different (*P* < 0.05), the subjects with GA genotype had lower serum TC, HDL-C and ApoAI levels than the subjects with AA genotype. When serum lipid parameters in Han were stratified according to sex, we showed that the levels of TC, HDL-C and ApoAI in females but not in males were different among the AA, GA and GG genotypes (*P* < 0.05), the participants with GA genotype had lower serum TC, HDL-C and ApoAI levels than the participants with AA genotype.

**Table 3:** The genotypes of rs3757354 SNP and serum lipid levels in the Bai Ku Yao and Han populations

**Genotype**	**n**	**TC (mmol/L)**	**TG (mmol/L)**	**HDL-C (mmol/L)**	**LDL-C (mmol/L)**	**ApoAI (g/L)**	**ApoB (g/L)**	**ApoAI/ ApoB**
Bai Ku Yao								
AA	160	4.38±1.16	1.06(0.66)	1.67±0.47	2.58±0.97	1.31±0.35	0.84±0.24	1.72±0.88
GA	308	4.36±0.87	1.00(0.76)	1.68±0.38	2.55±0.70	1.33±0.30	0.85±0.23	1.70±0.77
GG	159	4.42±0.78	0.96(0.57)	1.76±0.45	2.60±0.71	1.36±0.34	0.84±0.21	1.77±0.79
*F*	–	0.150	2.437	3.206	0.162	2.052	0.354	1.241
*P*	–	0.861	0.296	0.041	0.851	0.129	0.702	0.290
Han Chinese								
AA	122	4.97±1.01	1.15(0.74)	1.93±0.49	2.70±0.81	1.47±0.29	0.91±0.21	1.69±0.46
GA	293	4.72±1.05	1.02(0.78)	1.81±0.44	2.66±0.81	1.40±0.25	0.90±0.24	1.65±0.50
GG	199	4.72±0.88	1.02(0.58)	1.92±0.52	2.64±0.68	1.43±0.29	0.88±0.20	1.70±0.58
*F*		4.635	2.494	3.871	0.644	3.394	1.070	0.347
*P*		0.010	0.287	0.021	0.526	0.034	0.343	0.707
Bai Ku Yao/male								
AA	75	4.44±1.50	1.17(0.82)	1.72±0.55	2.53±1.27	1.37±0.40	0.81±0.28	1.93±1.17
GA	161	4.35±0.94	1.11(0.96)	1.71±0.42	2.47±0.76	1.38±0.34	0.82±0.23	1.86±0.94
GG	67	4.52±0.85	1.11(0.74)	1.81±0.52	2.60±0.83	1.45±0.41	0.83±0.24	1.97±1.05
*F*	–	0.216	0.284	0.979	0.314	1.315	0.051	0.193
*P*	–	0.806	0.867	0.377	0.731	0.270	0.950	0.825
Bai Ku Yao/female								
AA	85	4.33±0.76	0.97(0.55)	1.62±0.39	2.62±0.59	1.26±0.29	0.86±0.21	1.53±0.43
GA	147	4.37±0.79	0.93(0.59)	1.66±0.33	2.64±0.62	1.27±0.25	0.88±0.23	1.53±0.48
GG	92	4.34±0.71	0.85(0.47)	1.73±0.39	2.60±0.61	1.30±0.26	0.84±0.19	1.62±0.48
*F*		0.016	3.755	1.833	0.144	0.734	0.508	1.125
*P*		0.984	0.153	0.162	0.866	0.481	0.602	0.326
Han/male								
AA	61	4.90±0.91	1.27(0.99)	1.84±0.43	2.62±0.72	1.42±0.23	0.90±0.22	1.66±0.43
GA	142	4.74±1.04	1.03(0.79)	1.83±0.47	2.65±0.81	1.41±0.26	0.89±0.23	1.67±0.50
GG	91	4.66±0.90	1.08(0.75)	1.83±0.49	2.66±0.72	1.37±0.29	0.89±0.22	1.63±0.66
*F*	–	1.247	4.060	0.058	0.093	0.925	0.157	0.276
*P*	–	0.289	0.131	0.943	0.912	0.398	0.854	0.759
Han/female								
AA	61	5.03±1.10	1.03(0.76)	2.01±0.54	2.78±0.89	1.51±0.33	0.92±0.21	1.71±0.49
GA	151	4.70±1.06	1.00(0.74)	1.80±0.41	2.66±0.80	1.38±0.24	0.91±0.26	1.63±0.50
GG	108	4.76±0.86	0.99(0.49)	2.01±0.53	2.62±0.66	1.48±0.29	0.88±0.18	1.75±0.49
*F*	–	4.063	0.039	6.623	2.290	5.796	1.109	1.342
*P*	–	0.018	0.981	0.002	0.103	0.003	0.331	0.263

*TC*, total cholesterol; *TG*, triglyceride; *HDL-C*, high-density lipoprotein cholesterol; *LDL-C*, low-density lipoprotein cholesterol; *ApoAI*, apolipoprotein AI; *ApoB*, apolipoprotein B.

### Risk factors for serum lipid parameters

Multiple linear regression analysis showed that serum TG and HDL-C levels in Bai Ku Yao were correlated with the genotypes of rs3757354 SNP (*P* < 0.05 for each; Table 4), whereas the levels of TC in Han (Table 4), and TC, LDL-C in Han females (Table 5) were correlated with the genotypes (*P* < 0.05 for all).

**Table 4:** Correlation between the genotypes of rs3757354 SNP and serum lipid parameters in the Bai Ku Yao and Han populations

**Lipid parameter**	**Relative factor**	**Unstandardized coefficient**	**Std. error**	**Standardized coefficient**	***t***	***P***
Bai and Han						
TC	Body mass index	0.066	0.010	0.191	6.918	0.000
	Ethnic group	0.367	0.053	0.187	6.985	0.000
	Age	0.007	0.002	0.107	3.932	0.000
	Diastolic blood pressure	0.010	0.003	0.104	3.706	0.000
TG	Body mass index	0.064	0.011	0.163	5.699	0.000
	Gender	−0.244	0.067	−0.110	−3.660	0.000
	Genotype	−0.112	0.043	−0.072	−2.612	0.009
	Diastolic blood pressure	0.007	0.003	0.063	2.192	0.028
	Alcohol consumption	0.095	0.046	0.063	2.079	0.037
HDL-C	Ethnic group	0.184	0.025	0.200	7.276	0.000
	Alcohol consumption	0.122	0.019	0.196	6.295	0.000
	Age	0.004	0.001	0.131	4.811	0.000
	Body mass index	−0.017	0.004	−0.103	−3.766	0.000
	Cigarette smoking	−0.064	0.019	−0.105	−3.392	0.000
	Genotype	0.038	0.017	0.060	2.192	0.028
LDL-C	Body mass index	0.061	0.008	0.224	7.972	0.000
	Age	0.006	0.001	0.112	4.058	0.000
	Alcohol consumption	−0.130	0.029	−0.124	−4.465	0.000
	Diastolic blood pressure	0.006	0.002	0.082	2.834	0.004
ApoAI	Alcohol consumption	0.087	0.011	0.213	7.795	0.000
	Ethnic group	0.104	0.016	0.172	6.333	0.000
	Age	0.003	0.001	0.168	6.195	0.000
ApoB	Body mass index	0.019	0.002	0.233	8.445	0.000
	Age	0.002	0.000	0.124	4.547	0.000
	Diastolic blood pressure	0.003	0.001	0.126	4.432	0.000
	Ethnic group	0.045	0.012	0.098	3.620	0.000
	Alcohol consumption	−0.020	0.009	−0.065	−2.360	0.018
ApoAI/ApoB	Alcohol consumption	0.186	0.026	0.202	7.210	0.000
	Body mass index	−0.036	0.007	−0.153	−5.376	0.000
	Diastolic blood pressure	−0.004	0.002	−0.068	−2.382	0.017
Bai Ku Yao						
TC	Body mass index	0.085	0.015	0.218	5.597	0.000
	Age	0.009	0.003	0.132	3.392	0.000
TG	Alcohol consumption	0.217	0.075	0.142	2.888	0.004
	Body mass index	0.076	0.019	0.157	4.055	0.000
	Gender	−0.507	0.129	−0.219	−3.922	0.000
	Cigarette smoking	−0.201	0.079	−0.138	−2.552	0.010
	Genotype	−0.132	0.062	−0.081	−2.118	0.034
HDL-C	Alcohol consumption	0.092	0.022	0.165	4.200	0.000
	Age	0.003	0.001	0.111	2.837	0.004
	Genotype	0.051	0.023	0.087	2.212	0.027
LDL-C	Body mass index	0.071	0.013	0.219	5.616	0.000
	Age	0.007	0.002	0.120	3.023	0.002
	Alcohol consumption	−0.144	0.041	−0.140	−3.548	0.000
	Diastolic blood pressure	0.007	0.003	0.092	2.268	0.023
ApoAI	Alcohol consumption	0.119	0.016	0.279	7.289	0.000
	Age	0.002	0.001	0.101	2.649	0.008
ApoB	Body mass index	0.023	0.004	0.242	6.249	0.000
	Age	0.002	0.001	0.138	3.501	0.000
	Alcohol consumption	−0.041	0.012	−0.136	−3.491	0.000
	Diastolic blood pressure	0.003	0.001	0.108	2.685	0.007
ApoAI/ApoB	Alcohol consumption	0.288	0.041	0.271	7.071	0.000
	Body mass index	−0.053	0.013	−0.158	−4.133	0.000
Han						
TC	Body mass index	0.062	0.012	0.202	5.021	0.000
	Systolic blood pressure	0.007	0.002	0.122	2.969	0.003
	Age	0.006	0.003	0.101	2.550	0.011
	Genotype	−0.118	0.054	−0.085	−2.186	0.029
TG	Body mass index	0.053	0.014	0.162	3.913	0.000
	Diastolic blood pressure	0.010	0.004	0.105	2.525	0.011
	Cigarette smoking	0.153	0.060	0.100	2.522	0.011
HDL-C	Age	0.005	0.001	0.160	4.088	0.000
	Alcohol consumption	0.133	0.029	0.196	4.655	0.000
	Cigarette smoking	−0.103	0.029	−0.151	−3.546	0.000
	Body mass index	−0.018	0.006	−0.125	−3.194	0.001
	Gender	0.084	0.039	0.087	2.166	0.030
LDL-C	Body mass index	0.062	0.009	0.263	6.773	0.000
	Age	0.006	0.002	0.117	3.015	0.002
	Alcohol consumption	−0.148	0.045	−0.136	−3.264	0.001
	Cigarette smoking	0.140	0.045	0.127	3.073	0.002
ApoAI	Age	0.004	0.001	0.252	6.524	0.000
	Alcohol consumption	0.057	0.015	0.148	3.774	0.000
	Gender	0.059	0.021	0.108	2.763	0.005
ApoB	Body mass index	0.017	0.003	0.240	6.115	0.000
	Systolic blood pressure	0.002	0.001	0.153	3.802	0.000
	Age	0.002	0.001	0.106	2.705	0.007
ApoAI/ApoB	Body mass index	−0.031	0.006	−0.191	−4.823	0.000

*TC*, total cholesterol; *TG*, triglyceride; *HDL-C*, high-density lipoprotein cholesterol; *LDL-C*, low-density lipoprotein cholesterol; *ApoAI*, apolipoprotein AI; *ApoB*, apolipoprotein B.

**Table 5:** Relative factors for serum lipid parameters between males and females in both ethnic groups

**Lipid parameter**	**Relative factor**	**Unstandardized coefficient**	**Std. error**	**Standardized coefficient**	***t***	***P***
Bai Ku Yao/male						
TC	Body mass index	0.136	0.027	0.277	5.004	0.000
TG	Body mass index	0.137	0.038	0.204	3.611	0.000
HDL-C	Alcohol consumption	0.132	0.036	0.207	3.681	0.000
	Age	0.005	0.002	0.159	2.836	0.004
	Body mass index	−0.028	0.012	−0.131	−2.364	0.018
LDL-C	Body mass index	0.113	0.023	0.271	4.877	0.000
ApoAI	Alcohol consumption	0.134	0.028	0.271	4.883	0.000
	Age	0.003	0.001	0.126	2.273	0.023
ApoB	Body mass index	0.031	0.006	0.279	5.024	0.000
	Diastolic blood pressure	0.003	0.001	0.113	2.028	0.043
ApoAI/ApoB	Alcohol consumption	0.290	0.077	0.212	3.790	0.000
	Body mass index	−0.090	0.026	−0.196	−3.513	0.000
Bai Ku Yao/female						
TC	Age	0.011	0.003	0.192	3.511	0.000
	Body mass index	0.059	0.017	0.196	3.528	0.000
	Alcohol consumption	−0.205	0.090	−0.126	−2.275	0.023
TG	Alcohol consumption	0.207	0.075	0.151	2.742	0.006
	Body mass index	0.039	0.014	0.156	2.833	0.004
	Genotype	−0.108	0.046	−0.126	−2.337	0.020
LDL-C	Age	0.009	0.002	0.212	3.917	0.000
	Body mass index	0.050	0.013	0.211	3.823	0.000
	Alcohol consumption	−0.222	0.072	−0.169	−3.092	0.002
ApoB	Diastolic blood pressure	0.003	0.001	0.113	2.019	0.044
	Body mass index	0.015	0.005	0.179	3.259	0.001
	Age	0.003	0.001	0.166	2.957	0.003
ApoAI/ApoB	Diastolic blood pressure	−0.009	0.003	−0.185	−3.373	0.000
	Alcohol consumption	0.113	0.055	0.112	2.052	0.040
Han/male						
TC	Diastolic blood pressure	0.012	0.005	0.148	2.471	0.014
	Body mass index	0.037	0.017	0.128	2.139	0.033
	Age	0.007	0.004	0.122	2.137	0.033
TG	Diastolic blood pressure	0.019	0.006	0.179	2.999	0.002
	Body mass index	0.054	0.022	0.150	2.510	0.012
HDL-C	Alcohol consumption	0.186	0.036	0.300	5.166	0.000
	Age	0.004	0.002	0.145	2.610	0.009
	Cigarette smoking	−0.089	0.033	−0.157	−2.736	0.006
	Body mass index	−0.018	0.008	−0.131	−2.366	0.018
LDL-C	Body mass index	0.050	0.013	0.220	3.875	0.000
	Cigarette smoking	0.177	0.055	0.189	3.212	0.001
	Alcohol consumption	−0.139	0.060	−0.136	−2.300	0.022
ApoAI	Age	0.004	0.001	0.265	4.850	0.000
	Alcohol consumption	0.087	0.019	0.246	4.495	0.000
ApoB	Body mass index	0.014	0.004	0.215	3.822	0.000
	Age	0.002	0.001	0.168	2.976	0.003
ApoAI/ApoB	Alcohol consumption	0.151	0.041	0.209	3.668	0.000
	Body mass index	−0.026	0.009	−0.162	−2.851	0.004
Han/female						
TC	Body mass index	0.086	0.018	0.268	4.829	0.000
	Systolic blood pressure	0.008	0.003	0.144	2.618	0.009
	Genotype	−0.153	0.076	−0.108	−2.020	0.044
TG	Cigarette smoking	0.335	0.095	0.192	3.538	0.000
	Body mass index	0.048	0.015	0.171	3.160	0.001
HDL-C	Age	0.005	0.002	0.169	3.028	0.002
	Body mass index	−0.017	0.009	−0.111	−1.992	0.047
LDL-C	Body mass index	0.078	0.013	0.318	5.922	0.000
	Genotype	−0.124	0.058	−0.114	−2.145	0.032
	Age	0.006	0.003	0.109	2.060	0.040
ApoAI	Age	0.004	0.001	0.234	4.284	0.000
ApoB	Body mass index	0.020	0.004	0.279	5.308	0.000
	Systolic blood pressure	0.003	0.001	0.225	4.285	0.000
	Alcohol consumption	0.045	0.018	0.128	2.503	0.012
ApoAI/ApoB	Body mass index	−0.037	0.009	−0.234	−4.325	0.000
	Cigarette smoking	−0.119	0.053	−0.122	−2.254	0.024

*TC*, total cholesterol; *TG*, triglyceride; *HDL-C*, high-density lipoprotein cholesterol; *LDL-C*, low-density lipoprotein cholesterol; *ApoAI*, apolipoprotein AI; *ApoB*, apolipoprotein B.

Serum lipid parameters were also correlated with several environmental factors such as age, gender, BMI, alcohol consumption, cigarette smoking, blood pressure in both ethnic groups (*P* < 0.05-0.001; Table 4).

## Discussion

The present study shows that the levels of serum TC, HDL-C, LDL-C, ApoAI and ApoB were lower in Bai Ku Yao than in Han. There were no significant differences in TG levels and the ratio of ApoAI to ApoB between the two ethnic groups. It is well known that dyslipidemia is the result of a combination of genetic and environmental factors [6-9]. LDL-C, HDL-C and TG concentrations are strongly influenced by the genetic constitution of each individual. Bai Ku Yao is a special and isolated subgroup of the Yao minority in China. Strict intra-ethnic marriages have been performed in this population from time immemorial. Therefore, we believed that the genetic background and some lipid metabolism-related gene polymorphisms in this population may be different from those in Han nationality.

In the present study, we showed that the frequency G allele of rs3757354 SNP was lower in Bai Ku Yao than in Han. The frequencies of AA, GA and GG genotypes were also different between the two ethnic groups. There were no significant differences in the genotypic and allelic frequencies between males and females in both ethnic groups. These results indicated that the prevalence of the G allele variant of the rs3757354 SNP may have a racial/ethnic specificity.

The potential relationship between the MYLIP SNPs and plasma or serum lipid levels in humans has been evaluated in several previous GWAS. However, previous findings on the association of rs3757354 SNP with the changes in plasma lipid levels were inconsistent. MYLIP in GWAS has been shown significant associations with LDL-C concentrations in Europeans [15-17]. Jeemon *et al*. [49] identified several novel loci including rs3757354 SNP associated with LDL-C in the implications of discoveries from genome-wide association studies. Teslovich *et al*. [23], Waterworth *et al*. [25] and Weissglas-Volkov *et al*. [35] also demonstrated that rs3757354 SNP was genome-wide significant association with LDL-C. Furthermore, Banka *et al*. [37] and Kararigas *et al*. [38] found that MYLIP is a sex-specific element influencing contractile function. However, Santos *et al*. [36] showed MYLIP p.N342S polymorphism was not associated with lipid profiles in the Brazilian population. In the present study, we showed that the levels of HDL-C in Bai Ku Yao were different among the three genotypes, the G allele carriers had higher serum HDL-C levels than the G allele noncarriers. The levels of TC, HDL-C and ApoAI in Han were also different among the three genotypes, the subjects with GA genotype had lower serum TC, HDL-C and ApoAI levels than the subjects with AA genotype. These findings were restricted to females but not males. Multiple linear regression analysis also showed that serum TG and HDL-C levels in Bai Ku Yao were correlated with the genotypes of rs3757354 SNP, whereas the levels of TC in Han, and TC, LDL-C in Han females were associated with the genotypes. These findings suggest that the association of MYLIP rs3757354 SNP and serum lipid levels is different between the two ethnic groups. There may be a sex-specific association of rs3757354 SNP and serum lipid parameters in Han population.

It is well known that environmental factors such as dietary patterns, lifestyle, obesity, physical inactivity, and hypertension are all strongly related with serum lipid levels [6,7]. Furthermore, exposure to different lifestyle and environmental factors in our population resident in Guangxi may further modify the effect of genetic variation on serum lipid phenotypes. Multivariate linear regression analysis showed that age, sex, BMI, alcohol consumption, cigarette smoking, and blood pressure were involved in determining serum lipid parameters. These findings suggest that the environmental factors also play an important role in determining serum lipid levels in our study populations. Differences in serum lipid levels between the two ethnic groups could be related to factors such as differences in the genetic background, dietary patterns and lifestyle factors and their interactions. Although Bai Ku Yao and Han reside in the same region, there were significant differences in their diet and lifestyle. Corn was the staple food and rice, soy, buck wheat, sweet potato, and pumpkin products were the subsidiary foods in Bai Ku Yao. Approximately 90% of the beverages were corn wine and rum. The alcohol content is about 15% (v/v). They are also accustomed to drink Hempseed soup. In contrast, rice was the staple food and corn, broomcorn, potato, and taro products were the subsidiary foods in Han. About 90% of the beverage was rice wine. The content of alcohol is about 30% (v /v). The staple and subsidiary foods are more favorable for serum lipid profiles in Bai Ku Yao than in Han. Corn contains abundant dietary fiber and plant protein [50]. Dietary fiber can decrease serum TC levels [51]. Plant protein can promote the transportation and excretion of free cholesterol. Dietary soy protein has well documented beneficial effects on serum lipid concentrations [52,53].

## Conclusion

The present study shows that there were significant differences in the genotypic and allelic frequencies of MYLIP rs3757354 SNP between Bai Ku Yao and Han populations. The levels of HDL-C in Bai Ku Yao were different among the genotypes, the G allele carriers had higher serum HDL-C levels than the G allele noncarriers. The levels TC, HDL-C and ApoAI in Han were different among the genotypes, the participants with GA genotype had lower serum TC, HDL-C and ApoAI levels than the participants with AA genotype. The levels of TG and HDL-C in Bai Ku Yao were correlated with the genotypes, whereas the levels of TC in Han, and TC, LDL-C in Han females were associated with the genotypes. These results suggest that the association of MYLIP rs3757354 SNP and serum lipid levels is different between the two ethnic groups. There may be a sex-specific association of rs3757354 SNP and serum lipid parameters in the Han population.

## Competing interests

The authors declare that they have no competing interests.

## Authors’ contributions

TTY participated in the design, undertook genotyping, performed the statistical analyses, and drafted the manuscript. RXY conceived the study, participated in the design, carried out the epidemiological survey, collected the samples, and helped to draft the manuscript. QL, PH, XNZ, KKH, DFW and LHHA carried out the epidemiological survey and collaborated to the genotyping. All authors read and approved the final manuscript.
